# Eigenvector centrality and its variability over time are promising indicators of alterations in brain function due to early amyloid deposition

**DOI:** 10.1093/braincomms/fcad104

**Published:** 2023-04-06

**Authors:** Stavros Skouras

**Affiliations:** Department of Neurology, Inselspital University Hospital Bern, Bern, CH-3010, Switzerland; Department of Fundamental Neurosciences, Faculty of Medicine, University of Geneva, Geneva, CH-1202, Switzerland; Department of Biological and Medical Psychology, University of Bergen, Bergen, NO-5020, Norway; machineMD AG, Bern, CH-3008, Switzerland

## Abstract

This scientific commentary refers to ‘Eigenvector centrality dynamics are related to Alzheimer’s disease pathological changes in non-demented individuals’, by Lorenzini *et al.* (https://doi.org/10.1093/braincomms/fcad088).

**This scientific commentary refers to ‘Eigenvector centrality dynamics are related to Alzheimer’s disease pathological changes in non-demented individuals’, by Lorenzini *et al.* (https://doi.org/10.1093/braincomms/fcad088).**

Alzheimer’s disease is a pernicious neurodegenerative disease sustained by convoluted biological processes that have posed a centenarian scientific and medical challenge. Prior to the onset of readily observable symptoms such as memory decline, cognitive impairment, and neurodegeneration, a lengthy process of tacit disease incubation precedes, which can often last several decades. This incubational phase appears to be asymptomatic, with the main exception of abnormal accumulation levels for certain proteins in the brain, which in recent years have become detectable using positron emission tomography, as well as by proxy based on the analysis of cerebrospinal fluid or blood samples.

According to the predominant scientific framework for clinical research on Alzheimer’s disease,^1^ a typical case first presents with aggregation of amyloid-β peptide 42 (amyloid-β_42_) into plaques in the neuropil of the neocortex, characterizing a state of increased risk for developing Alzheimer’s disease. Elevated amyloid-β_42_ levels are considered to trigger, or at least to increase the likelihood for, phosphorylated tau (p-tau) accumulation into neurofibrillary tangles that collect inside neurons. The combination of elevated levels for both amyloid-β_42_ and p-tau in the brain, in the absence of cognitive impairment, characterizes the preclinical stage of Alzheimer’s disease. As time passes, preclinical Alzheimer’s disease progresses to hippocampal atrophy that results in memory decline and eventually to wider neurodegeneration that results initially in Mild Cognitive Impairment and later in dementia.

The socioeconomic burden imposed on national healthcare systems by dementia, poses a significant threat to their sustainability beyond present times. Due to this reason, Alzheimer’s disease research has united public and private stakeholders across academia and the pharmaceutical industry. In the European Union, this was best encapsulated by the project EPAD (www.ep-ad.org) that was co-funded by the Innovative Medicines Initiative (www.imi.europa.eu). EPAD is an acronym for European Prevention of Alzheimer’s Dementia and its consortium comprised the largest public-private partnership in Alzheimer’s disease research, resulting in a 59 million euro project that ran for 5 years between 2015 and 2020. EPAD aimed to implement a large scale, continuous, adaptive clinical trial with multiple agents, across multiple arms, to streamline the testing and development of preventative treatments for Alzheimer’s disease. EPAD recruited more than 2000 research participants into a longitudinal cohort study, involving the inter-sectoral collaboration of 39 institutions and organisations, led by Janssen Pharmaceutica and the University of Edinburgh. EPAD aimed to facilitate the development of effective therapies for Alzheimer’s disease, by creating a large, pan-European cohort of research participants at high risk for Alzheimer’s disease, with complete neuropsychological, neuroimaging and neurobiological, Open Access datasets.^2,3^

In the present issue of *Brain Communications*, Lorenzini *et al*.^4^ have utilised 701 resting-state fMRI (rs-fMRI) datasets from the EPAD database, in an innovative way, ultimately to address the novel question of whether changes in Eigenvector Centrality (EC) variability across time are informative of early Alzheimer’s disease stages. To do this, Lorenzini *et al*.^4^ followed a standard analysis procedure involving typical preprocessing steps, Eigenvector Centrality Mapping (ECM) and permutation-based correction for multiple comparisons with threshold-free cluster enhancement. For each participant, ECM was performed 100 times in sliding windows, each comprised of 100 functional volumes, giving rise to dynamic measures of EC variability across time. From the 701 rs-fMRI datasets included, 469 participants were amyloid-negative (A-; i.e. presenting amyloid-β_42_ levels within the normal, healthy range) and 232 were amyloid-positive (A+; i.e. presenting elevated, abnormal levels of amyloid-β_42_). Out of the amyloid-positive participants, 181 presented a high risk for Alzheimer’s disease, characterized by abnormal levels of amyloid-β_42_ but normal levels of p-tau (A+T-) and 51 participants presented preclinical Alzheimer’s disease, characterized by abnormal levels of both amyloid-β_42_ and p-tau (A+T+). Lorenzini *et al*.^4^ compared the ECM results between the amyloid-positive and amyloid-negative groups, as well as between participants at risk for Alzheimer’s disease (A+T-) and participants with preclinical Alzheimer’s disease (A+T+).

Of methodological interest, to investigate the temporal dynamics of Eigenvector Centrality (EC), Lorenzini *et al*.^4^ applied an innovative analytical approach, with regards to the use of dual regression on the output of their dynamic ECM, while controlling the effect of potentially confounding variables. As a side note, EC originated in social network theory and it was originally developed to represent the comparative influence of network nodes in a social network. At the turn of the century, Google outperformed its competitors by using EC as the key measure to rank its search engine results. One decade ago, Wink *et al*.^5^ and Lohman *et al*.^6^ independently published two Open Access software tools that enabled the application of EC to neuroimaging (github.com/amwink/bias/tree/master/matlab/fastECM; github.com/lipsia-fmri/lipsia). Nowadays, in functional neuroimaging, the EC of a brain region is generally considered as a direct measure of the level to which that region is a functional connectivity hub, having rigorously considered all implicit functional connectivity patterns across the entire brain.

The key findings produced by Lorenzini *et al*.^4^ show the following (see Table 1 for statistical significance levels).

1. During the earliest phase of amyloid deposition, widespread differences in EC are evident in the human brain. Overall, amyloid-positive participants presented lower EC across the right precuneus, the posterior parietal lobule, the medial and ventral portions of the inferior and middle occipital lobe, as well as higher EC in mediofrontal regions extending to left anterior-temporal areas and the cingulate.
2. The latter higher EC coincides with a significant reduction of EC variability over time in the same frontotemporal/cingulate brain areas. Moreover, this lower EC variability was already present at the earliest stage of amyloid deposition, in the group at risk for Alzheimer’s disease (A+T-) and was even stronger in the group with preclinical Alzheimer’s disease (A+T+), compared to the amyloid-negative controls.
3. There were neither significant differences in EC, nor EC variability over time, between the group at risk for Alzheimer’s disease (A+T-) and the group with preclinical Alzheimer’s disease (A+T+). This is a crucial finding because it suggests that the main changes in EC occur during the earliest stage of amyloid deposition, in participants at risk for Alzheimer’s disease, prior to the preclinical stage.
4. Significant reductions in EC variability over time were identified in the amyloid-positive group for the default mode network (DMN) and the visual resting-state network (RSN).
5. Cognitive performance correlated negatively with dynamic EC variability in the DMN and visual RSN only in the amyloid-positive group and not in the amyloid-negative group.

**Table 1:** Overview of statistical significance levels for the key findings by Lorenzini *et al*.^4^

	A+ versus A-	A+T- versus A-T-	A+T+ versus A+T-	A-	A+
EC in parietal cluster (incl. PCu, PPL)	* P_corr._ < 0.05	* P_corr._ < 0.05	n.s.	-	-
EC in occipital cluster	* P_corr._ < 0.05	* P_corr._ < 0.05	n.s.	-	-
EC in frontotemporal/cingulate cluster	* P_corr._ < 0.05	* P_corr._ < 0.05	n.s.	-	-
EC variability in parietal cluster (incl. PCu, PPL)	n.s.	-	-	-	-
EC variability in occipital cluster	n.s.	-	-	-	-
EC variability in frontotemporal/cingulate cluster	** P < 0.005	* P < 0.05	n.s.	-	-
EC variability in DMN	*** P < 0.001	-	-	-	-
EC variability in visual network	**** P < 0.0001	-	-	-	-
Negative correlation of MMSE score with EC variability in DMN	-	-	-	n.s. *P* = 0.66	* P < 0.05
Negative correlation of RBANS_VCI_ with EC variability in DMN	-	-	-	n.s. *P* = 0.58	* P < 0.05
Negative correlation of RBANS_VCI_ with EC variability in visual network	-	-	-	n.s. *P* = 0.53	** P < 0.01
Negative correlation of RBANS_IMI_ cognitive performance with EC variability in visual network	-	-	-	n.s. *P* = 0.58	** P < 0.01

Missing entries are not applicable or not reported in the original manuscript.

Abbreviations: A- ∼ Amyloid negative; A+ ∼ Amyloid positive; T- ∼ Tau negative; T+ ∼ Tau positive; EC ∼ Eigenvector Centrality; dEC ∼ dynamic Eigenvector Centrality; PCu ∼ Precuneus; PPL ∼ Posterior Parietal Lobule; DMN ∼ Default Mode Network; MMSE ∼ Mini-Mental State Examination; RBANS ∼ Repeatable Battery for the Assessment of Neuropsychological Status; VCI ∼ Visuo-Constructional Index; IMI ∼ Immediate Memory Index; corr. ∼ corrected for multiple comparisons; n.s. ∼ not significant; * ~ p<0.05; ** ~ p<0.01; *** ~ p<0.001; **** ~ p<0.0001.

In summary, widespread changes in EC occurred in participants at risk for Alzheimer’s disease, earlier than the preclinical stage of Alzheimer’s disease, including reductions in dynamic EC variability across major RSNs, correlating with cognitive performance. In this light and in relation to previous evidence,^7^ these findings provide strong support to the assertion that EC-related changes in the early stages of the pathophysiological continuum of Alzheimer’s disease, are intimately connected to mechanisms of functional compensation. In fact, it appears that during the earliest phase of amyloid deposition, prior to preclinical Alzheimer’s disease, maintenance of good cognitive performance is supported by lower EC variability in a widespread frontotemporal/cingulate network that exhibits both higher and more stable EC compared to amyloid-negative controls. The combination of both higher and more stable EC in the amyloid-positive group, points to a ceiling effect occurring on the neural connectivity capacity of these frontotemporal/cingulate regions (the concept of neural capacity was originally formulated as a subcomponent of neural reserve, referring to the maximal activation level that a brain area or network involved in a task can reach and sustain as the cognitive demands of that task keep increasing^8^). Because the significant negative correlation between EC variability and cognitive performance was not observed in the amyloid-negative control group and cognitive performance did not differ significantly between the amyloid-positive and amyloid-negative groups, it is reasonable to interpret the combined increased EC and increased EC stability as a maximal plateau of EC; i.e. the frontotemporal/cingulate areas reach a sustained EC peak to maintain cognitive performance levels unaffected by the amyloid deposition for as long as possible.

An independent interpretation is proposed by Lorenzini *et al*.,^4^ who suggest that dynamic connectivity is impaired by amyloid deposition, through a circular mechanism whereby amyloid deposition is both the cause and the result of neuronal activity changes. It is highly probable that both interpretations are true at the same time. It is undisputed that amyloid deposition exerts a disruptive effect on global brain function and connectivity. At the same time, it is also established that mechanisms of functional compensation (such as, e.g. hippocampal hyperactivity^9^) manifest during the early stages of Alzheimer’s disease. Currently, there is no evidence regarding how sustainable such patterns of hyperactivation or hyperconnectivity would be over long periods of time in the range of years, and whether the neurobiological processes required to sustain maximal EC in the observed areas could gradually contribute to further amyloid deposition.

EC and dynamic EC variability across numerous brain networks, provide a rich set of sophisticated metrics that likely reflect both processes of functional degradation, as well as concurrent processes of functional compensation. In a similar previous study together with colleagues, we proposed that across the pathophysiological continuum of Alzheimer’s disease, decreases in EC reflect degrading brain function while concurrent increases in EC, particularly in the cingulate, reflect counterparting compensatory processes.^7^ Remarkably, the cingulate also appears as a local maximum in the main results by Lorenzini *et al*.,^4^ replicating observations from the most similar previous studies and corroborating a role for EC differences in the cingulate as a marker of progression across the entire pathophysiological continuum from health to dementia^7,9,10^ (Fig. 1). Interestingly, healthy ageing appears to be characterized by the opposite effect, i.e. decreased EC in the cingulate cortex,^9,11^ offering some promise for differentiating Alzheimer’s disease risk from healthy ageing. Moreover, the cingulate is a key brain area of a recently discovered task-invariant cognitive reserve network.^12^

**Figure 1:**
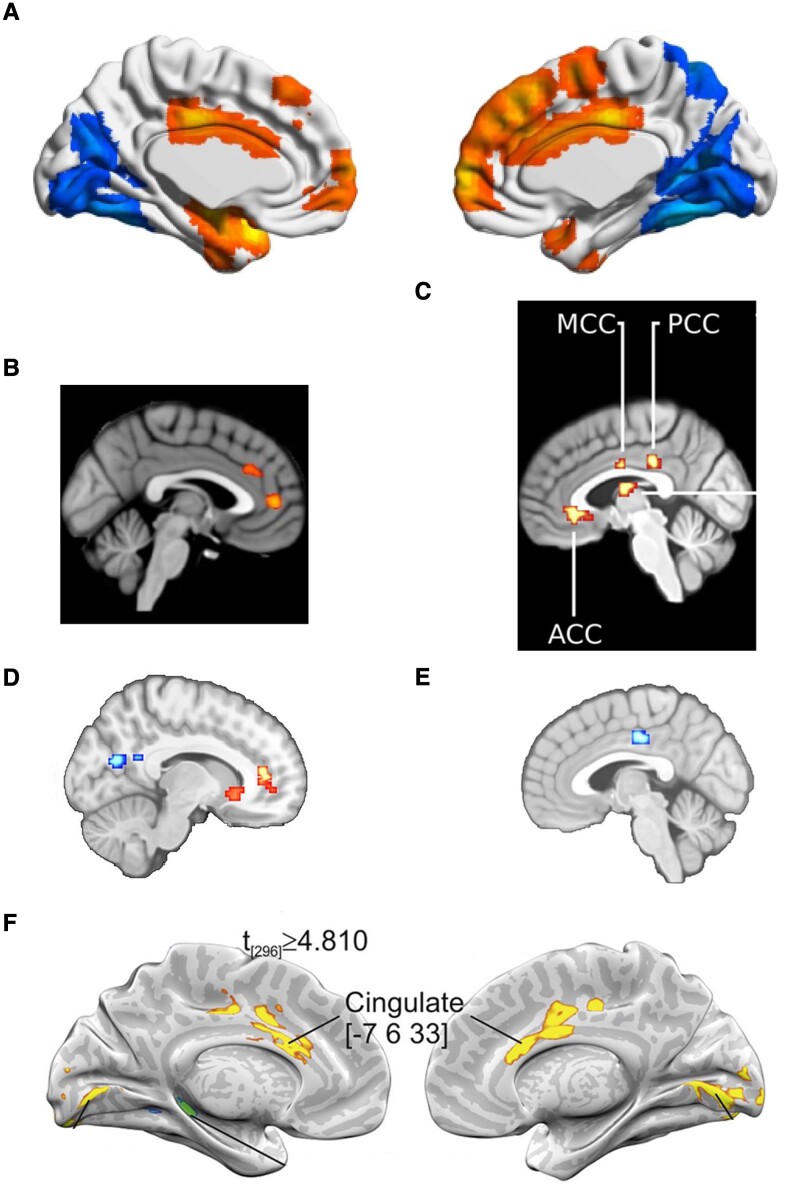
**Overview of replicated EC findings in the cingulate, in relation to Alzheimer’s disease.** (**A**) Significantly higher EC in posterior and middle cingulate cortex in participants at risk for Alzheimer’s disease, compared to age-matched controls, during resting-state (*N* = 608).^4^ Reprinted from Lorenzini *et al*.^4^ (**B**) Significantly higher EC in anterior cingulate cortex in Alzheimer’s patients with MCI, compared to age-matched controls, during resting state (*N* = 82).^10^ Reprinted from Binnewijzend *et al*.^10^ with permission from John Wiley Inc. © 2013 Wiley Periodicals, Inc. (**C**) Significant positive correlation between CSF biomarker levels from health to dementia in older adults and EC in anterior (ACC), middle (MCC) and posterior cingulate cortex (PCC), during resting-state (*N* = 96).^7^ Reprinted from Skouras *et al*.^7^ with permission from Elsevier. © 2019 The Authors. (**D**) Significant positive correlation between EC in anterior cingulate cortex and CSF p-tau levels, in older adults, during hippocampal downregulation (*N* = 48).^9^ Reprinted from Skouras *et al*.^9^ by permission of Oxford University Press. ©2020 The Author(s). (**E**) Significant negative correlation between EC in middle cingulate cortex and healthy ageing (controlled for biomarkers and Alzheimer’s disease risks), in older adults, during hippocampal downregulation (*N* = 48).^9^ Reprinted from Skouras *et al*.^9^ by permission of Oxford University Press. ©2020 The Author(s). (**F**) Significantly lower EC in middle and anterior cingulate cortex in healthy older adults, compared to young controls (*N* = 60).^11^ Reprinted from Antonenko *et al*.^11^ with permission from Elsevier. © 2018 Elsevier Inc.

As implied by Lorenzini *et al*.,^4^ EC-related metrics could indeed potentially serve as functional neuromarkers of Alzheimer’s disease risk and progression, with high value for applications in disease prognosis and in monitoring the therapeutic efficacy of pharmaceutical and non-pharmaceutical interventions. To achieve this, as well as to fully explain the observed effects, it is necessary to conduct similar analyses using multiyear longitudinal data. Points of caution for future analyses regard data harmonization and balancing the distribution of participants in each group across the different imaging sites. Moreover, as for all connectivity-based analyses, ECM will benefit from the use of multislice acquisition techniques for subsecond whole-brain volume repetition times, resulting in the acquisition of longer timeseries within a given time duration. This directly translates to more datapoints during an fMRI scanning session and ECM results with higher statistical power, which is crucial to enhance sensitivity and replicability for the development of clinically useful biomarkers.

In conclusion, Lorenzini *et al*.^4^ have contributed very important novel insights from an unprecedented, landmark multicentric study. Their findings demonstrate that changes in EC, as well as EC variability over time, are implicated in the neuro-functional manifestation of the earliest stages of Alzheimer’s disease. Commendably, the analysis of Lorenzini *et al*.^4^ is both innovative as well as based on Open Access data and Open Source software, comprising a prime example of effective Open Science practices.

## Data Availability

No new data were created or analysed for this article.
